# Elevated CO_2_ Modifies N Acquisition of *Medicago truncatula* by Enhancing N Fixation and Reducing Nitrate Uptake from Soil

**DOI:** 10.1371/journal.pone.0081373

**Published:** 2013-12-05

**Authors:** Huijuan Guo, Yucheng Sun, Yuefei Li, Xianghui Liu, Qin Ren, Keyan Zhu-Salzman, Feng Ge

**Affiliations:** 1 State Key Laboratory of Integrated Management of Pest Insects and Rodents, Institute of Zoology, Chinese Academy of Sciences, Beijing, People’s Republic of China; 2 University of Chinese Academy of Sciences, Beijing, People’s Republic of China; 3 Jining Normal College, Inner Mongolia Autonomous Region, Jining, People’s Republic of China; 4 Department of Entomology, Texas A&M University, College Station, Texas, United States of America; Centro de Investigación y de Estudios Avanzados del IPN, Mexico

## Abstract

The effects of elevated CO_2_ (750 ppm vs. 390 ppm) were evaluated on nitrogen (N) acquisition and assimilation by three *Medicago truncatula* genotypes, including two N-fixing-deficient mutants (*dnf1-1* and *dnf1-2*) and their wild-type (Jemalong). The proportion of N acquisition from atmosphere and soil were quantified by ^15^N stable isotope, and N transportation and assimilation-related genes and enzymes were determined by qPCR and biochemical analysis. Elevated CO_2_ decreased nitrate uptake from soil in all three plant genotypes by down-regulating nitrate reductase (*NR*), nitrate transporter *NRT1.1* and NR activity. Jemalong plant, however, produced more nodules, up-regulated N-fixation-related genes and enhanced percentage of N derived from fixation (%Ndf) to increase foliar N concentration and N content in whole plant (Ntotal Yield) to satisfy the requirement of larger biomass under elevated CO_2_. In contrast, both *dnf1* mutants deficient in N fixation consequently decreased activity of glutamine synthetase/glutamate synthase (GS/GOGAT) and N concentration under elevated CO_2_. Our results suggest that elevated CO_2_ is likely to modify N acquisition of *M. truncatula* by simultaneously increasing N fixation and reducing nitrate uptake from soil. We propose that elevated CO_2_ causes legumes to rely more on N fixation than on N uptake from soil to satisfy N requirements.

## Introduction

Global atmospheric CO_2_ concentrations have been increasing at an accelerating rate [1]. The concentration, which was 280 ppm before industrialization and was 394 ppm in December 2012 (Mauna Loa Observatory: NOAA-ESRL), is expected to reach at least 550 ppm by the year 2050 [1]. The effects of elevated CO_2_ on C3 plants are generally characterized by increased photosynthesis, growth and yield in plant tissues [2]. Under elevated CO_2_, the “extra C” is assimilated and transported from leaves and shoots to roots, and the C:N ratio is consequently increased [3]. Thus, plant responses to elevated CO_2_ are likely to be limited by the availability of N.

Besides increases in biomass and productivity, a common characteristic of non-leguminous C3 plants in an elevated CO_2_ environment is a 10–15% decrease in N concentration (g of N per g of plant tissue ) [4]. Three major hypotheses have been proposed to explain this phenomenon [5]. According to the reduced uptake hypothesis, N content is reduced because decreased stomatal conductance and transpiration under elevated CO_2_ reduces N uptake by roots [6]. The N loss hypothesis presumes that N losses increase under elevated CO_2_ because of increasing NH_3_ volatilization or increasing root exudation of organic N [7]. The dilution hypothesis, which has received the most attention, considers that N content is diluted under elevated CO_2_ by accumulation of more total non-structural carbohydrates (TNC), which results in a greater biomass for a given quantity of N [8]. Depending on the species or genotype, these hypotheses may partially or largely explain the substantial reduction in the N content in non-leguminous plants under elevated CO_2_ [9]. Furthermore, elevated CO_2_ has little effect on the N content in legumes, which might be attributed to their unique ability to utilize atmospheric N_2_ [4], but it still lacks the experimental evidence to address the physiological mechanism underlying N metabolism of legume plants under elevated CO_2._

Leguminous plants acquire N by three major pathways. First, legumes uptake ammonia (NH_4_^+^) from soil and incorporate it into organic compounds. Second, legumes uptake nitrate from soil and reduce it to NH_4_^+^. Third, legumes in symbioses with N-fixing bacteria can obtain N from the atmosphere by N fixation, i.e., by converting N_2_ to NH_4_^+^ [10]. Among these three pathways, N fixation is most costly in terms of energy and resources. LaRue and Patterson (1981), for example, found that four legumes including *Glycine max*, *Vigna unguiculata*, *Phaseolus vulgaris*, and *Pisum sativum*, consume an average of 6.7 g of carbohydrate to obtain 1 g of N by symbiosis [11]. Acquiring N via uptake of nitrate or ammonia from soil required less carbohydrate C than acquiring N by symbiosis [12], [13]. Nitrogenase activity, the most important enzyme involved in N fixation, and nodule formation are often suppressed when nitrate or ammonia availability is sufficient to meet the requirements of plant growth [14]. Thus, it seems that legume plants preferentially obtain N via uptake from the soil rather than fixation from the atmosphere [13].

To sustain and maximize growth and biomass under elevated CO_2_, legumes require additional N [8]. Owing to the high C consumption required for N fixation, elevated CO_2_ helps legumes fix N from atmosphere [15]. After reviewing 127 studies, Lam *et al.,* (2012) concluded that the amount of N fixed from the atmosphere by legumes increased 38% under elevated CO_2_, which was accompanied by increases in whole plant nodule number (+33%), nodule mass (+39%), and nitrogenase activity (+37%) [16]. Furthermore, enhancement of N fixation in legumes is essential for overcoming the N limitation under elevated CO_2_ [17]. However, the relative contributions of N fixation and uptake from soil to the N content of legumes under elevated CO_2_ are largely unknown. It is likely that legumes adjust their means of utilizing N resources to adapt to environmental changes [18], and a CO_2_-enriched environment may affect the crosstalk between the different N acquisition pathways in legumes.

The current study examined N acquisition via N fixation and N uptake in N-fixing-deficient mutants *(dnf1*) and wild-type (Jemalong) of *M. truncatula*. We tested the hypothesis that *M. truncatula* plants regulate the relative contribution of N fixation and N uptake from soil to maximum the N assimilation rate to satisfy the higher N requirement under elevated CO_2_. The specific objectives were to determine: (1) how elevated CO_2_ affects N fixation from the atmosphere and N uptake from soil; and (2) whether elevated CO_2_ affects N assimilation of the *M. truncatula* genotypes. To help meet these objectives, we measured the expression of key genes and the activity of key enzymes involved in N acquisition and assimilation (glutamine synthase/glutamate synthase, GS/GOGAT cycle) [19]. Meanwhile, ^15^N stable isotope technique was used to determine N acquisition and partitioning, and estimate the proportion of N fixed from atmosphere/N uptake from soil [20].

## Materials and Methods

### Atmospheric CO_2_ Concentration Treatments

This experiment was performed in eight octagonal open-top field chambers (OTCs) (4.2 m diameter and 2.4 m height) at the Observation Station of the Global Change Biol Group, Institute of Zoology, Chinese Academy of Science in Xiaotangshan County, Beijing, China (40°11′N, 116°24′E). The atmospheric CO_2_ concentration treatments were: (1) current atmospheric CO_2_ levels (390 µl/L), and (2) elevated CO_2_ levels (750 µl/L, the predicted level in about 100 years) (IPCC, 2007). Four blocks were used, and each block contained one OTC with ambient CO_2_ and one with elevated CO_2_. From seedling emergence to the harvesting of *M. truncatula* plants (27 August to 15 October 2011, a total of 50 days), CO_2_ concentrations were monitored and adjusted with an infrared CO_2_ analyzer (Ventostat 8102, Telaire Company, Goleta, CA, USA) once every minute to maintain relatively stable CO_2_ concentrations. The measured CO_2_ concentrations throughout the experiment (mean ± SD per day) were 391±23 ppm in the ambient CO_2_ chambers and 743±32 ppm in the elevated CO_2_ chambers. The auto-control system for maintaining the CO_2_ concentrations, as well as specifications for the OTCs, is detailed in Chen and Ge (2005) [21]. The tops of the OTCs were covered with nylon net to exclude insects. Air temperatures were measured three times per day throughout the experiment and did not differ significantly between the two treatments (24.9±3.4°C in OTCs with ambient CO_2_ vs. 26.2±3.9°C in OTCs with elevated CO_2_).

### M. Truncatula Mutants and Rhizobium Inoculation

Three *M. truncatula* genotypes were studied: the N-fixation-deficient mutants *dnf1-1* and *dnf1-2* as well as their wild-type Jemalong. These three genotypes were obtained from the laboratory of Sharon Long, Department of Biology, Stanford University. The nodules of these *dnf1* mutants are small and white and are blocked at an intermediate stage of development [22]. The *dnf1-1* mutant allele has a large deletion of at least 20 kb around TC121074 locus, and the *dnf1-2* mutant allele has an independent disruption of the TC121074 locus [20]. Although both mutants can be infected in the inner cortex, both lack acetylene reduction activity and *Nodulin31* expression and have only a small level of *nifH* expression in the symbiotic nodule [23].

After seeds were chemically scarified and surface sterilized by immersion in concentrated H_2_SO_4_ for 5 min, they were rinsed with sterilized water several times. The seeds were placed in Petri dishes filled with 0.75% agar, kept in the dark at 4°C for 2 days, and then moved to 25°C for 2 days to germinate. The germinated seeds were sown on sterilized soil and inoculated 2 days later with the bacterium *Sinorhizobium meliloti* Rm1021 [23], which was kindly provided by Professor Xinhua Sui (Department of Microbiology, College of Biological Sciences, Chinese Agricultural University). *S. meliloti* was cultured on YM (H_2_O 1000 ml, yeast 3 g, mannitol 10 g, KH_2_PO_4_ 0.25 g, K_2_HPO_4_ 0.25 g, MgSO_4_·7H_2_O 0.1 g, NaCl 0.1 g, pH 7.0–7.2) for 3 days at 28°C to obtain an approximate cell density of 10^8^ ml^−1^. At sowing, each seedling was inoculated with 0.5 ml of this suspension. After they had grown in sterilized soil for 2 weeks, the *M. truncatula* seedlings were individually transplanted into plastic pots (35 cm diameter and 28 cm height) containing sterilized loamy field soil (organic carbon 75 g/kg; N 500 mg/kg; P 200 mg/kg; K 300 mg/kg) and placed in OTCs on 27 August 2011. Each OTC contained 30 plants (10 each per genotype) with 240 plants in total.

Plants were maintained in the OTCs for 50 days. Pot placement was re-randomized within each OTC once every week to avoid any effects from the position of pots in each OTC. No chemical fertilizers and insecticides were used. Water was added to each pot once every 2 days.

### Plant Sampling and Preparation

All the plants of *M. truncatula* were randomly harvested on 13–15 October 2011. Root of each plant were carefully removed from soil and washed. A stereomicroscope was used to count the nodules on the entire root system of 6 plants from each *M. truncatula* genotype per OTC ( = 24 plants from each genotype at each CO_2_ level and 144 in total). After nodules were counted, the shoots and roots of each plant were collected, oven-dried (65°C) for 72 h, and weighed. The leaves and root tissues were then ground to a fine powder (approx. 0.85 mm size) and analyzed for total non-structural carbohydrates (TNCs), N concentration and ^15^N isotopic analysis. Another three plants from each *M. truncatula* genotype per OTC (9 plants per OTC and 72 plants in total) were randomly selected for enzyme analysis and real-time PCR. 50 mg of mature leaves and 100 mg of lateral roots from each plant were stored in freezing tubes at −75°C until used for real-time PCR. 0.5 g of mature leaves and 1.0 g of lateral roots from the same plants were frozen for enzyme analysis as described in the following paragraph.

### TNCs, N Concentration and δ^15^N Analysis

TNCs, mainly starch and sugars, in leaves and roots were quantified by acid hydrolysis following the method of Tissue & Wright (1995). N concentrations in leaves and roots were measured by Kjeltec N analysis (Foss automated Kjeltec™ instruments, Model 2100) [24].

δ^15^N were determined from approximately 3 mg plant sample with an isotope-ratio mass spectrometer (IRMS; Delta^plus^ XP and Delta C prototype Finnigan MAT, respectively, Finnigan MAT, Bremen, Germany; 0.1‰ precision). The *δ* ^15^N values represent nitrogen isotopic composition of the sample relative to that of atmospheric dinitrogen in ‰:
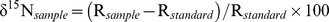



Where R*_standard_* is the ^15^N/ ^14^N ratio of atmospheric N_2_ and R*_sample_* is the^15^N/ ^14^N ratio of the sample plant. The repeated measurement precision was 0.2‰.

*Percentage of N derived from atmosphere and N uptake from soil*

Percentage of N fixed from atmosphere is a yield-independent parameter and was calculated according to Pausch *et al.,* (1996) [25]:
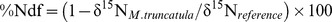



Where % Ndf is the percentage of N derived from atmosphere. δ^15^N*_M. truncatula_* is the δ^15^N of wild-type Jemalong, δ^15^N*_reference_* is the average value of δ^15^N of *dnf1-1* and *dnf1-2* in the same OTC. *dnf1-1* and *dnf1-2* have similar N uptake and rooting patterns as Jemalong but are deficient in nitrogen fixation, and therefore served as the reference plant for analyzing the N fixation of wild-type *M. truncatula*.

In order to evaluate changes in N source (i.e. as derived from N-fixation or soil) for *M. truncatula* plants, Nf Yield (N derived from N-fixation per plant) and Ns Yield (N derived from soil per plant) of Jemalong estimates were calculated as follows:
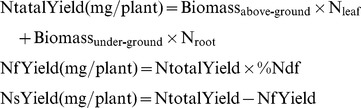



Where Ntotal Yield is the N content in per whole plant, Biomass_above-ground_ is the biomass of above-ground tissue in *M. truncatula* plants, Biomass_under-ground_ is the biomass of under-ground tissue, N_leaf_ is the N concentration of leaves and N_root_ is the N concentration of root.

### Activities of Enzymes Involved in N Uptake and Assimilation

The activities of nitrate reductase (NR), glutamine synthetase (GS), and glutamate synthase (GOGAT) in leaves and roots were determined using frozen tissue (approximately 0.5 g leaf tissue and approximately 1.0 g root tissue per plant). Once the tissue was ground to a fine powder, leaves or roots from three plants of the same genotype within each OTC were combined to form one sample from each OTC. The unit of replication for statistical analyses was the OTC (n = 4). An extract was obtained by grinding each leaf sample or root sample in 50 mM Tris HCl buffer (pH 7.8, 3 ml/g of leaf tissue) containing 1 mM MgCl_2_, 1 mM EDTA, 1 µM β-mercaptoethanol, and 1% (w/v) polyvinylpolypyrro-lidone. This extract was immediately frozen for later use. For assays, the thawed extract was centrifuged at 13,000 *g* for 10 min, and the enzyme activities were measured in the supernatant as described by Geiger *et al*. (1998) for NR [26], by Glévarec *et al*. (2004) for GS [27], and by Suzuki *et al*. (2001) for GOGAT [28]. Protein concentrations of leaves and roots were measured using bovine serum albumin as a standard. One unit (*U*) of GS/GOGAT activities are defined as the amount of the GS or GOGAT that catalyzes 1 nmol of glutamine or glutamate per minute in the homogenate.

### Expression of Genes Associated with N Fixation, Uptake, and Assimilation as Determined by Quantitative RT-PCR

Each treatment combination was replicated four times for biological repeats, and each biological repeat contained three technical repeats. The RNAeasy Mini Kit (Qiagen) was used to isolate total RNAs from *M. truncatula* leaves and roots, and 1 µg of RNA was used to generate the cDNAs. The mRNAs of the following nine target genes were quantified by real-time quantitative PCR: early nodule-specific protein 40 (*ENOD*) (maintenance of nodule symbiosis) [29], nodulation gene (*nodF*) (*nodF* genes are required for nodulation) [29], nitrogen-fixing gene (*nifH*) (*nifH* genes control synthesis of nitrogenase) [30], nitrate transporter *NRT1.1* (*NT*) [31], nitrate reductase (*NR*), ammonium transporter protein (*AMT*) [32], glutamine synthetase 2 (*GS*) [33], and glutamate synthetase (*GOGAT*) [33] (Figure S1 in File S3). Specific primers for each gene were designed from the *M. truncatula* EST sequences using PRIMER5 software (Table S1 in File S1). The PCR reactions were performed in 20 µL reaction volumes that included 10 µL of 2×SYBRs Premix EX TaqTM (Qiagen) master mix, 5 mM of each gene-specific primer, and 1 µL of cDNA template. Reactions were carried out on the Mx 3500P detection system (Stratagene) as follows: 2 min at 94°C; followed by 40 cycles of 20 s at 95°C, 30 s at 56°C, and 20 s at 68°C; and finally one cycle of 30 s at 95°C, 30 s at 56°C, and 30 s at 95°C. This PCR protocol produced the melting curves, which can be used to judge the specificity of PCR products. A standard curve was derived from the serial dilutions to quantify the copy numbers of target mRNAs. *β*-actin and *pnp* were used as internal qPCR standards for the analysis of plant and bacterial gene expression, respectively [29]. The relative level of each target gene was standardized by comparing the copy numbers of target mRNA with copy numbers of *β*-actin or *pnp* (the house-keeping gene), which remain constant under different treatment conditions. The levels of *β*-actin or *pnp* mRNAs in the control were examined in every PCR plate to eliminate systematic error. The fold-changes of target genes were calculated using the 2^−ΔΔCt^ normalization method.

### Statistical Analysis

Statistical analyses were performed with SPSS 13.0 software (SPSS Inc., Chicago, IL). Two-way analyses of variance (ANOVA) were used to analyze the effect of CO_2_ and plant genotype on *M. truncatula* growth traits, TNC, N concentration, Ntotal Yield and enzyme activities. If an ANOVA was significant, Tukey’s multiple range test was used for mean separation (*P*<0.05). Significance of the effect of CO_2_ on %Ndf, Nf Yield, Ns Yield of Jemlaong and genes regulating N metabolism were determined by independent *t*-tests.

## Results

### Plant Biomass and Nodule Number

**Figure 1:**
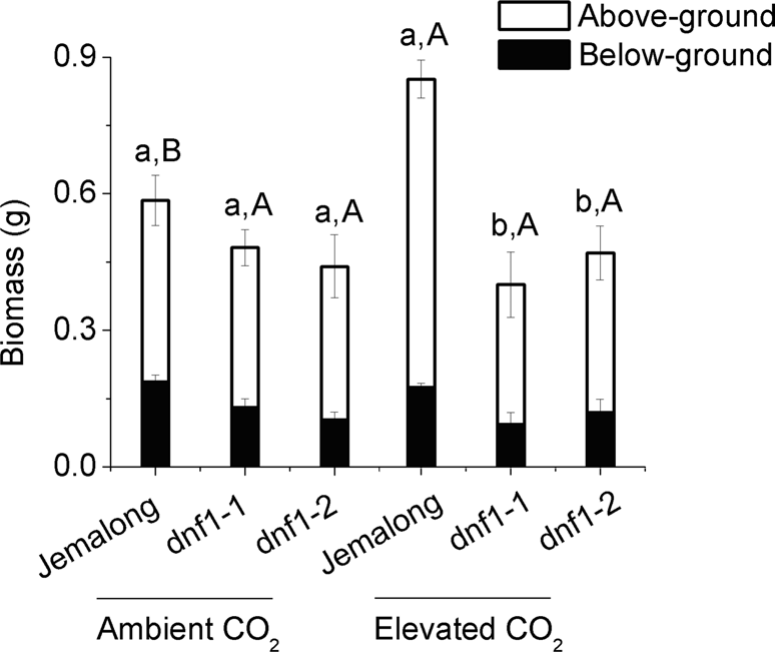
Above- and below-ground biomass of *M. truncatula* plants as affected by CO_2_ level and plant genotype: *dnf1-1* and *dnf1-2* are deficient in N fixation, and Jemalong is their wild type. Each value represents the average (±SE) of four replicates. Different lowercase letters indicate significant differences between ambient CO_2_ and elevated CO_2_ within the same genotype. Different uppercase letters indicate significant differences among genotypes within the same CO_2_ treatment as determined by Tukey’s multiple range test at *P*<0.05.

**Figure 2:**
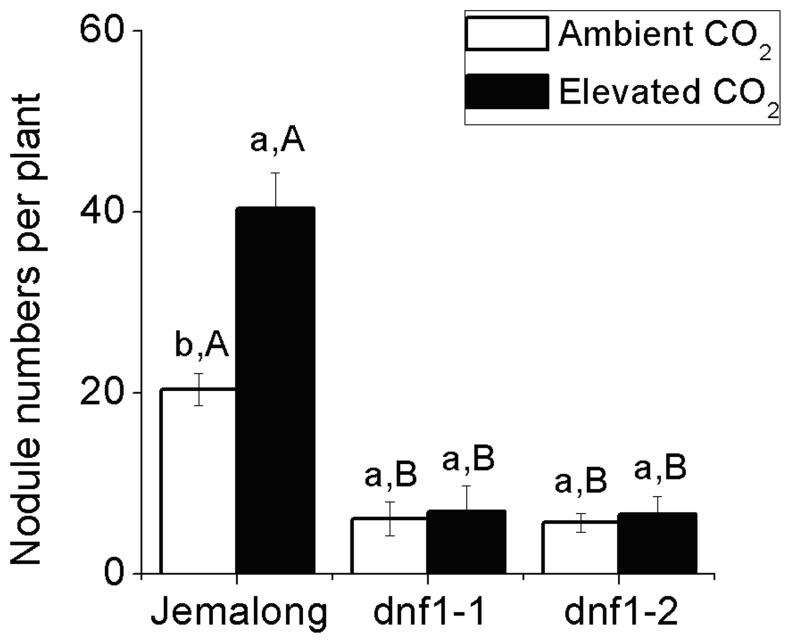
Nodule number per root of *M. truncatula* plant as affected by CO_2_ level and plant genotype: *dnf1-1* and *dnf1-2* are deficient in N fixation, and Jemalong is their wild type. Each value represents the average (±SE) of four replications. Different lowercase letters indicate significant differences between ambient CO_2_ and elevated CO_2_ within the same genotype. Different uppercase letters indicate significant differences among *M.* genotypes within the same CO_2_ treatment as determined by Tukey’s multiple range test at *P*<0.05.

CO_2_ level, genotype and their interaction significantly affected the above-ground biomass, below-ground biomass and total biomass (Table S2 in File S2). Total biomass did not significantly differ among the genotypes under ambient CO_2_ but was greater for the wild-type Jemalong than for the mutants under elevated CO_2_ (Fig. 1). In response to elevated CO_2_, above-ground biomass increased 37.1% and total biomass increased 41.9% for Jemalong plants but the biomass of *dnf1* mutant plants was not significantly affected by the CO_2_ treatments (Fig. 1). CO_2_ level and genotype significantly affected the nodule numbers (Table S2). Regardless of CO_2_ level, nodule number was greater for Jemalong than for the *dnf1* mutants (Fig. 2). Elevated CO_2_ increased nodule numbers of Jemalong but not of the mutants.

### TNC and N Characteristic in Plant

**Figure 3:**
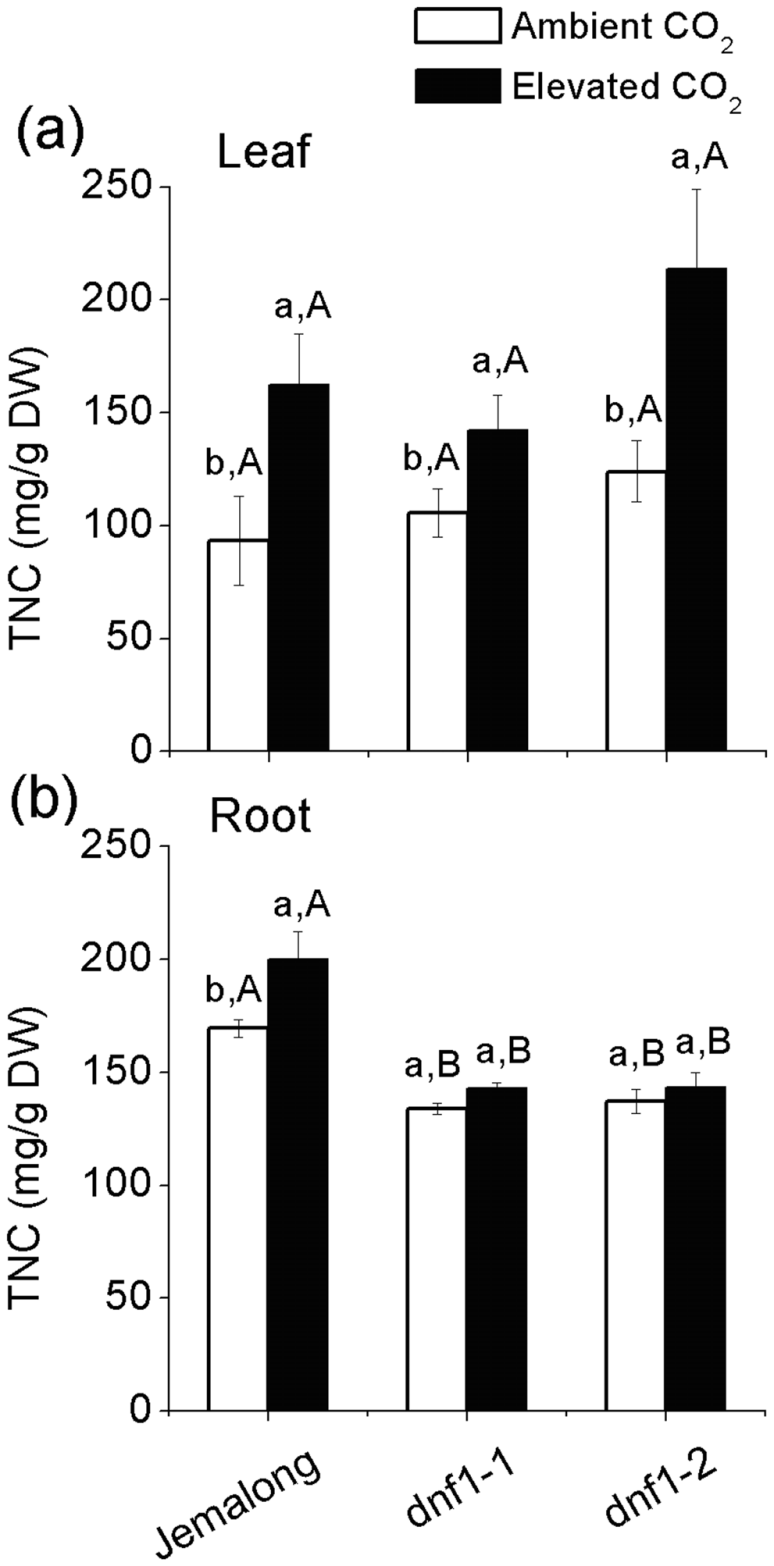
Total non-structural carbohydrate (TNC) content in leaves and roots of *M. truncatula* plants as affected by CO_2_ level and plant genotype: *dnf1-1* and *dnf1-2* are deficient in N fixation, and Jemalong is their wild type. Each value represents the average (±SE) of four replicates. Different lowercase letters indicate significant differences between ambient CO_2_ and elevated CO_2_ within the same genotype. Different uppercase letters indicate significant differences among genotypes with the same CO_2_ treatment as determined by Tukey’s multiple range test at *P*<0.05.

CO_2_ level significantly affected the foliar TNC, and all factors significantly affected the root TNC (Table S2). Elevated CO_2_ increased the TNC content in leaves and roots of Jemalong but only in leaves of *dnf1-1* and *dnf1-2* (Fig. 3). Foliar TNC content did not differ among the three *M. truncatula* genotypes (Fig. 3). Regardless of CO_2_ level, Jemalong had the highest root TNC content (Fig. 3).

**Figure 4:**
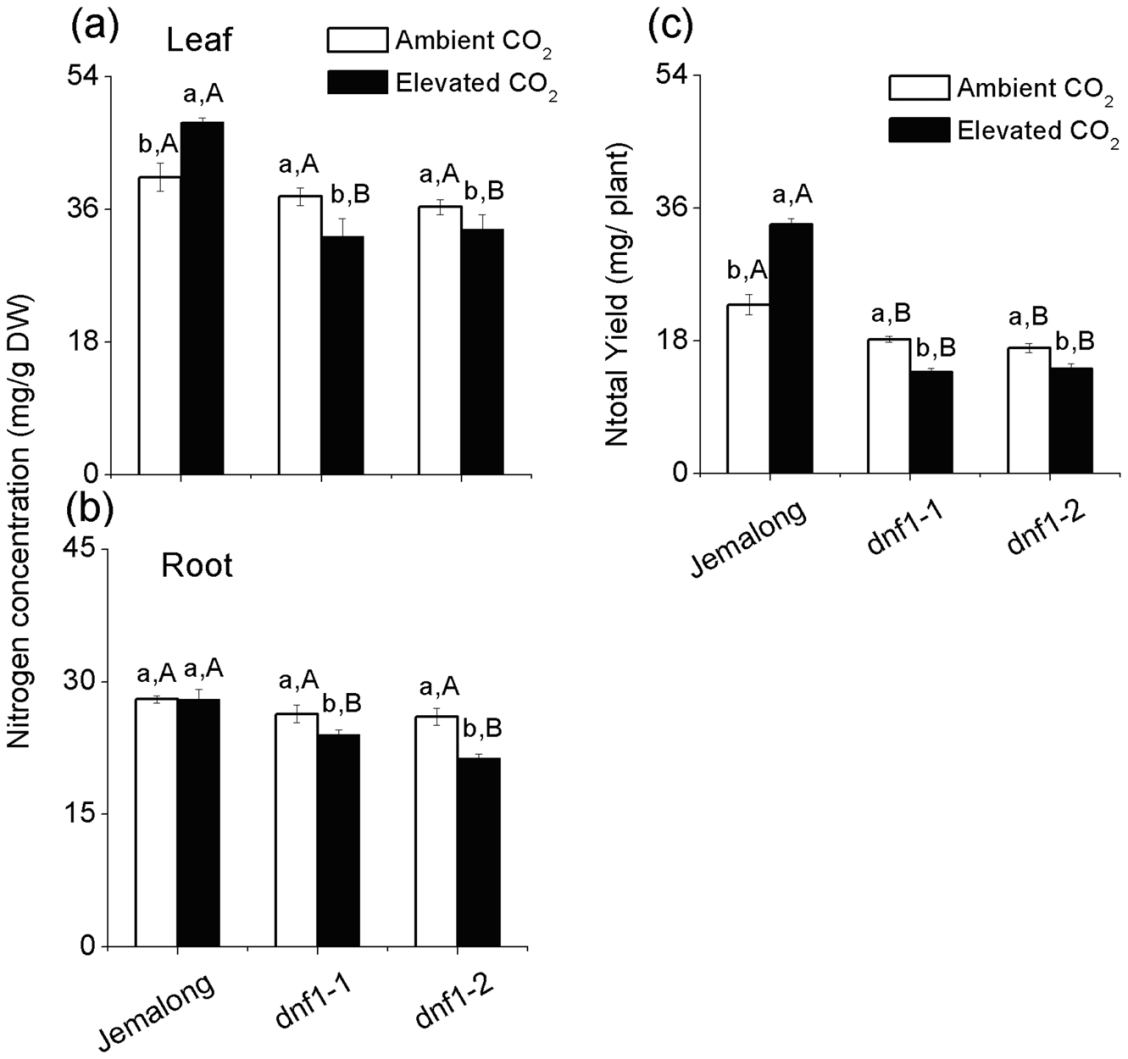
N concentrations in leaves and roots as well as Ntotal Yield (N content in per plant) of *M. truncatula* plants as affected by CO_2_ level and plant genotype: *dnf1-1* and *dnf1-2* are deficient in N fixation, and Jemalong is their wild type. Each value represents the average (±SE) of four replicates. Different lowercase letters indicate significant differences between ambient CO_2_ and elevated CO_2_ within the same genotype. Different uppercase letters indicate significant differences among genotypes within the same CO_2_ treatment as determined by Tukey’s multiple range test at *P*<0.05.

Genotype was significant for the foliar N concentration, and all factors significantly affected the root N concentration and Ntotal Yield (Table S2). Elevated CO_2_ increased the foliar N concentration and Ntotal Yield in Jemalong but reduced in both *dnf1* mutants (Fig. 4). Elevated CO_2_ reduced N concentration in the roots of both *dnf1* mutants but not in Jemalong (Fig. 4). Under ambient CO_2_, foliar N and root N concentrations were not significantly different among three genotypes. Under elevated CO_2_, however, N concentration in leaves and roots were higher in Jemalong than in the mutants (Fig. 4). Regardless of CO_2_ level, Jemalong had higher Ntotal Yield than *dnf1-1* and *dnf1-2* mutants (Fig. 4). Furthermore, elevated CO_2_ increased %Ndf and Nf Yield but decreased Ns Yield of Jemalong (Table 1).

**Table 1:** CO_2_ effects on N characteristics (%Ndf, Nf Yield, Ns Yield) of wild-type Jemalong plant.

	Jemalong		
	%Ndf^1^	Nf Yield^2^ (mg/plant)	Ns Yield^3^ (mg/plant)
Ambient CO_2_ (390 ppm)	39.8±0.7 b	9.1±0.7 b	13.8±0.4 a
Elevated CO_2_ (750 ppm)	65.3±1.2 a	25.3±1.1 a	11.5±1.0 b

Each value represents the average (±SE) of four replicates. Different lowercase letters indicate significant differences between ambient CO_2_ and elevated CO_2_ as determined by independent *t-*test at *P*<0.05.

1 percentage of N derived from atmosphere.

2 N derived from N-fixation per plant.

3 N derived from soil per plant.

### Activities of the Enzymes NR, GS and GOGAT

**Figure 5:**
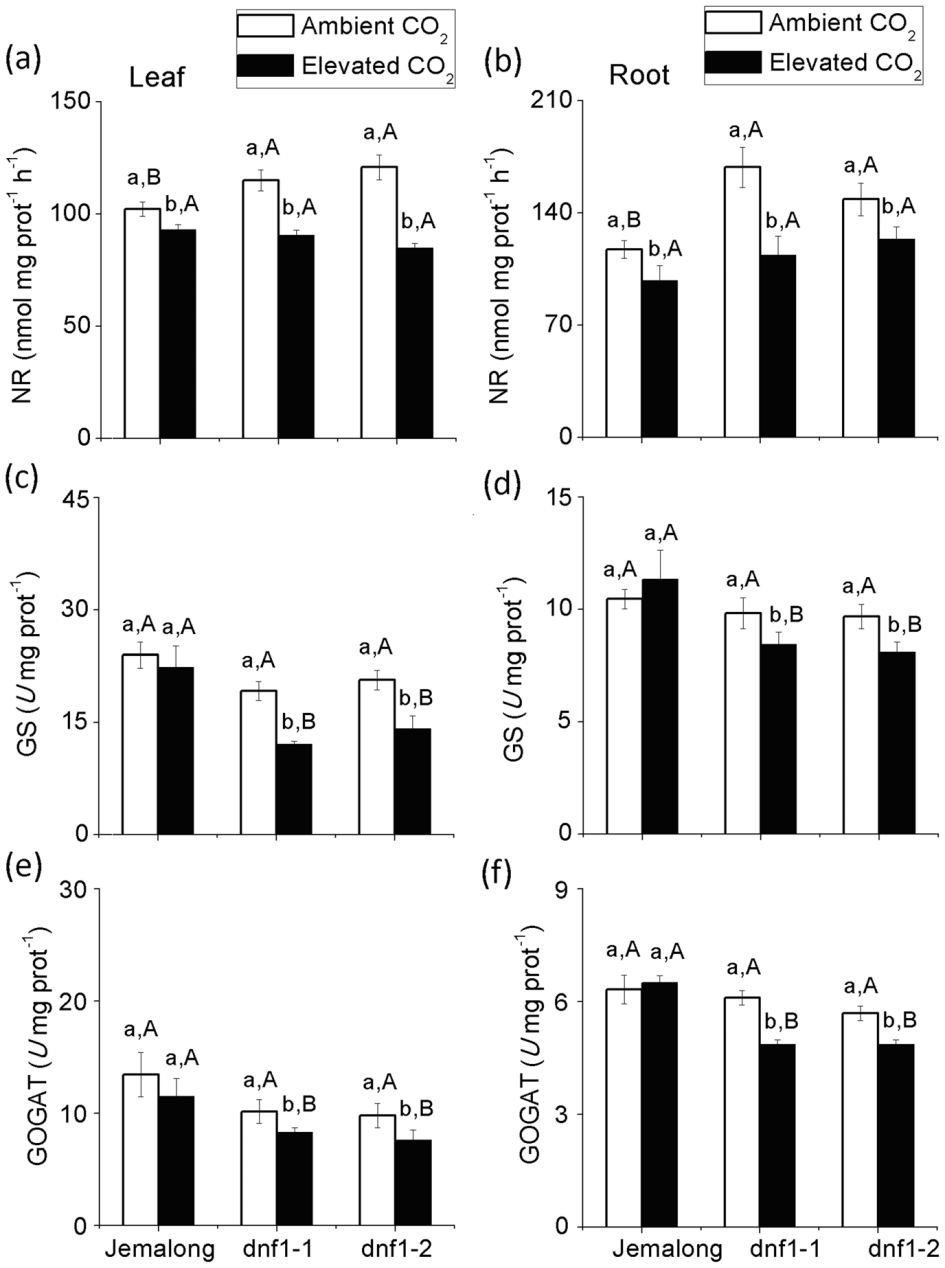
Activities of the enzymes involved in N reduction (NR) and in N assimilation (GS and GOGAT) in the leaves and roots of *M. truncatula* plants as affected by CO_2_ level and plant genotype: *dnf1-1* and *dnf1-2* are deficient in N fixation, and Jemalong is their wild type. Each value represents the average (±SE) of four replicates. Different lowercase letters indicate significant differences between ambient CO_2_ and elevated CO_2_ within the same genotype. Different uppercase letters indicate significant differences among genotypes within the same CO_2_ treatment as determined by Tukey’s multiple range test at *P*<0.05.

CO_2_ level was significant for the activities of foliar NR and root NR. (Table S2). Elevated CO_2_ reduced NR activity in the leaves and roots of all three genotypes (Table S2, Fig. 5). Under ambient CO_2_, NR activity in both leaves and roots were higher in both *dnf1* mutants than in Jemalong (Fig. 5). Under elevated CO_2_, however, NR activity did not differ among the three genotypes (Fig. 5).

Genotype and the interaction between CO_2_ and genotype significantly affected the foliar GS and root GS. All factors significantly affected the foliar GOGAT and root GOGAT (Table S2). Elevated CO_2_ decreased GS and GOGAT activities in the two *dnf1* mutants but not in Jemalong (Fig. 5). GS and GOGAT activities in Jemalong leaves and GOGAT in roots were higher than *dnf1-1* and *dnf1-2* mutant in both CO_2_ levels. GS activity in roots did not differ among the three genotypes under ambient CO_2_ but were higher in Jemalong than in the mutants under elevated CO_2_ (Fig. 5).

### Expression of Genes Associated with N Fixation, Uptake, and Assimilation as Determined by Quantitative RT-PCR

**Figure 6:**
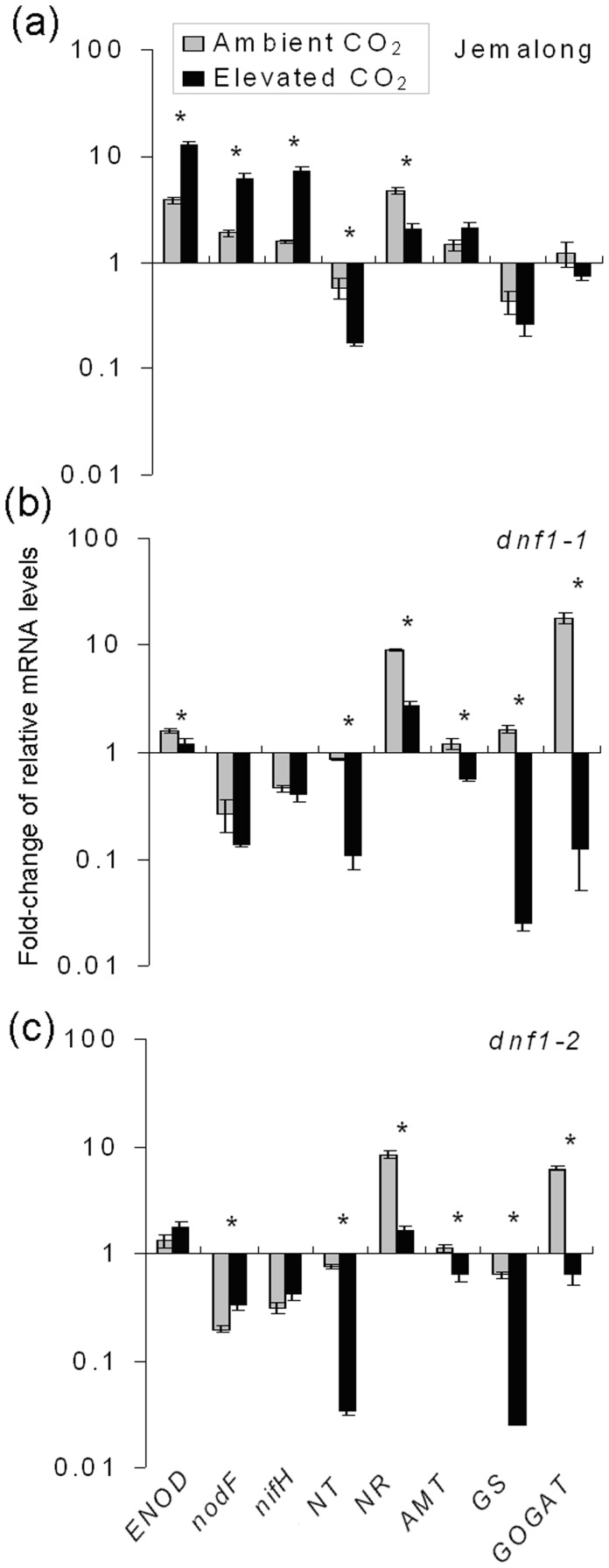
Expression of genes involved in N fixation (*ENOD*, *nodF*, and *nifH*), nitrate transportation and reduction (*NT* and *NR*), ammonium uptake (*AMT*), and N assimilation (*GS* and *GOGAT*) in leaves of *M. truncatula* plants as affected by CO_2_ level and plant genotype: *dnf1-1* and *dnf1-2* are deficient in N fixation, and Jemalong is their wild type. Values indicate fold-change in expression based on qPCR determination, and each value represents the average (±SE) of four replicates. An asterisk above a column indicates a significant difference in gene expression under ambient vs. elevated CO_2_ (*P*<0.05).

Elevated CO_2_ up-regulated the expression of N fixation related genes including *ENOD*, *nodF*, and *nifH*, but down-regulated the expression of nitrate uptake and transport related genes including *NR* and *NT* in Jemalong plants (Fig. 6). For *dnf1-1* and *dnf1-2*, elevated CO_2_ down-regulated the gene expression of *NR* and *NT*, and ammonia transport related genes *AMT*, and N assimilation related gene including *GS* and *GOGAT* (Fig. 6).

## Discussion

The notion that elevated CO_2_ can increase plant biomass and TNC content in plant tissues is widely accepted [8]. Although this concept was further supported by the current report, our results also indicate that the key element in the increase in biomass of *M. truncatula* under elevated CO_2_ is the availability of N (Fig. S2). Using *dnf1-1* and *dnf 2* mutants, we demonstrated that *M. truncatula* is able to adjust different N partitioning pathways to ensure a sufficient N supply under ambient CO_2_. Elevated CO_2_, however, reduced N uptake from soil by suppressing N uptake related gene and increased the reliance on fixation of atmospheric N_2_.

N availability is one of the key factors limiting plant growth and production. Elevated CO_2_ stimulating plant growth would increase the N demand of plants [34]. The extent of the CO_2_ response at the plant level could consequently be limited by N availability [35], [36]. In current study, since elevated CO_2_ increased foliar N concentration and Ntotal yield (Table 1; Fig. 4), Jemalong plants were able to produce more biomass under elevated CO_2_ (Fig. 1). Moreover, although TNC content in leaves and roots of *dnf1-1* and *dnf1-2* mutants were increased (Fig. 3), N concentration in leaves and roots as well as the Ntotal yield of *dnf1* mutant plants were decreased by elevated CO_2_ (Table 1; Fig. 4). It may suggest that *dnf1* mutants were unable to provide sufficient N to support the enhancement of biomass under elevated CO_2_ (Fig. 1). Thus, our results demonstrated that the symbiotic N_2_ fixation provided legumes an incomparable advantage in producing larger amounts of biomass under elevated CO_2_ [37].

The results of the current study show that legumes are very flexible in their utilization of N from soil and atmosphere under ambient CO_2_. Although the *dnf1* mutants are unable to fix atmospheric N_2_, GS/GOGAT activities involved in N assimilation and N concentration in leaves and roots did not differ from those of the wild-type Jemalong under ambient CO_2_. As indicated by increased gene expression (*NR*, *NT*) and enzyme activities (NR) of essential components of the alternate N acquisition pathways (Table 1; Fig. 5, 6), the *dnf1* mutants compensated for the loss of N fixation by enhancing their uptake of N from soil under ambient CO_2_. However, the GS/GOGAT activities and N concentration in *dnf1* plants were lower than in Jemalong plants under elevated CO_2_ (Fig. 4, 5), which indicated that elevated CO_2_ limited the N availability for both *dnf1* mutants. Furthermore, Lüscher *et al.* (2000) found even in the high soil N treatment, ineffectively nodulating lucerne were unable to increase the N concentration and biomass under elevated CO_2_ [38]. Our results confirmed that soil N is insufficient to meet the increasing N demand of *M. truncatula* which can fully transform increased C assimilation into biomass [39].

The soil N availability appears to be suppressed by elevated CO_2_ for all three *M. truncatula* genotypes, as reflected in decreased Ns Yield in Jemalong and Ntotal Yield in both *dnf1* mutants under elevated CO_2_. Furthermore, the enzyme activity of NR and the expression of *NT* and *NR* genes of all three genotypes were also down-regulated by elevated CO_2_ (Fig. 5). This indicates that elevated CO_2_ suppresses N uptake of *M. truncatula* from soil. This is consistent with the the finding that N uptake from soil by *Trifolium repens* were decreased under elevated CO_2_ grown in a grassland ecosystem [40]. In addition, our results showed that elevated CO_2_ down-regulated *NR* and *NT* but was not significant for ammonia transporter *AMT* (Fig. 6). It seems that the decreases of N uptake from soil were mainly associated with the decreases of nitrate uptake rather than ammonia uptake.

The decreased nitrate uptake under elevated CO_2_ could be explained by two factors: lower soil N availability and plant NO_3_^−^ reduction. Elevated CO_2_ reduced the soil N availability by increasing N immobilization and denitrification in soil. For example, elevated CO_2_ increased microbial community composition in rhizosphere soil of white clover, and subsequently increased N immobilization into the expanded microbial biomass [41]. Additionally, elevated CO_2_ increased the emission of N_2_O from soil [42], and this increase of N loss caused decreases of nitrate availability in soil. On the other hand, lower plant photorespiration induced by elevated CO_2_ could decrease the nicotinamide adenine dinucleotide (NADH) [43], which provides the energy required to convert NO_3_^−^ to NO_2_^−^ in the cytoplasm of leaf mesophyll cells [44]. Moreover, elevated CO_2_ increased HCO_3_^−^, and in turn inhibited NO_2_^−^ transportation from cytosol into the chloroplast [45], which led to a decrease in plant nitrate reduction.

Insufficient soil N uptake was considered to be one of the reasons for the increased contribution of N_2_ fixation under elevated CO_2_ [39]. In agreement with higher foliar N concentration and Ntotal Yield in the Jemalong plants, there was a strong increase in the %Ndf, nodule numbers and up-regulation of N fixation-related genes (ENOD, *nodF* and *nifH*) under elevated CO_2_. Furthermore, elevated CO_2_ decreased N concentration and Ntotal Yield in both *dnf1* mutants, suggesting that the increased N concentration and Ntotal Yield in Jemalong was solely the result of elevated CO_2_-induced increases of N_2_ fixation. In addition, the fixation of N_2_ required substantial amount of C resource, and the respiration measurements showed the costs of C for N assimilation from nitrate seem to be lower than those for N_2_ fixation [14]. Elevated CO_2_, however, provided the sufficient C to satisfy the energy demand for N_2_ fixation, and decreased soil N availability under elevated CO_2_ accelerated N_2_ fixation in Jemalong plants [46].

Although elevated CO_2_ tends to increase the N concentration and modify N acquisition patterns of legumes, there is little evidence that elevated CO_2_ can affect the key enzymes involved in N assimilation [19]. GS and GOGAT are critical enzymes involved in the assimilation of ammonia, which is not only derived from nitrate reduction and N_2_ fixation but also from some secondary metabolism processes, i.e. photorespiration or amino acid catabolism [47]. Photorespiration is one of the most important physiological process in which high amounts of ammonium are released [48], which was likely to be suppressed by elevated CO_2_ [45]. This is probably the reason why GS and GOGAT activities were unaffected even though *M. truncatula* could acquire more N from fixation under elevated CO_2_. Furthermore, elevated CO_2_ decreased the enzyme activity and transcripts of GS and GOGAT in both *dnf1* mutants, and these decreases were accompanied by decreases in the N concentration of roots and leaves. Thus, it appears that *M. truncatula* and presumably other legumes require N fixation to maintain N assimilation under elevated CO_2_.

In conclusion, regardless of wild-type and N fixation mutant, elevated CO_2_ decreased N uptake from soil by down-regulating the expression of NR and NT of *M. truncatula*. Wild-type plants, however, are able to up-regulate N fixation related genes and increase nodule numbers under elevated CO_2_ to maintain sufficient N concentration for plant growth. This suggests that as atmospheric CO_2_ continues to rise, legumes may rely more on N fixation due to less on N uptake from soil. This could benefit agriculture because higher N fixation may compensate N depletion from soil, which would facilitate the growth of non-leguminous plants. Although our study has important implications for agriculture and for regional and global N budgets under predicted CO_2_ conditions, the enhancement of leguminous N fixation by elevated CO_2_ is environment-dependent [49]. N fixation can be limited by the availability of other soil nutrients (i.e., molybdenum, phosphorus, potassium) or by abiotic stresses (i.e., salinity, alkalinity, acidity, drought, fertilizer, metal toxicity) [50]. Moreover, since N uptake from soil is constrained by elevated CO_2_, legumes are very likely to find it more difficult to maintain their growth under elevated CO_2_ when they are subjected to stresses that reduce N fixation. Considering few studies have examined the interactive effects of elevated CO_2_ and other abiotic stress on the N dynamics of legume, environmental variables in addition to atmospheric CO_2_ concentrations should be considered when predicting future N dynamics of legumes. Besides, Understanding the N dynamics of legume plants and ensuring food security in the future also require a deeper understanding of interaction between legume plants and other organisms such as herbivorous insects.

## Supporting Information

File S1 **Table S1: Primer sequences used for real-time quantitative PCR.**
(DOC)

File S2 **Table S2:** ***P*** **values from two-way ANOVAs for the effects of CO_2_ level,** ***M. truncatula*** **genotype, and their interaction on the growth traits and foliar chemical components of alfalfa plants.**
(DOC)

File S3 **Figure S1: The legume genes shown in this figure were tracked in the current study and are involved in N fixation, N uptake from soil, and N assimilation as indicated.** The genes include: early nodule-specific protein 40 (*ENOD*), nodulation genes (*nodF*), nitrogen-fixing genes (*nifH*), nitrate transporter NRT1.1 (*NT*), nitrate reductase (*NR*), nitrate transporter NRT1.1 (*NT*), ammonium transporter protein (*AMT*), glutamine synthase 2 (*GS2*), and glutamate synthase (*GOGAT*).
(DOC)
