# Supplementary material for: Elevated CO2 Modifies N Acquisition of Medicago truncatula by Enhancing N Fixation and Reducing Nitrate Uptake from Soil
Source: PLoS One. 2013 Dec 5;8(12):e81373. doi: 10.1371/journal.pone.0081373 (PMC3855279; doi:10.1371/journal.pone.0081373)
Supplement: File S1 — Table S1: Primer sequences used for real-time quantitative PCR. (DOC) [file pone.0081373.s001.doc]

**Supplementary Tables**

**Table S1.** Primer sequences used for real-time quantitative PCR.

| Gene | Primer sequence（5’-3’） | Function | Sampled from |
| --- | --- | --- | --- |
| *ENOD* | *F*: CTTCCCCACTACCTTTCTAT  *R*: ATTATTTGCTTCTGCTACTC | Nodule-formation related | Root |
| *nodF* | *F:* CATTGGCATCATCAAGAACCG  *R:* TAAATCGCCGACTATTAACGCG | Nodule-formation related | Rhizobia |
| *nifH* | *F:* CGGCTTTGCGATGCCTATT  *R:* GCATAGAGCGCCATCATCTCA | Nodule-formation related | Rhizobia |
| *NT* | *F*: AAAGACGGAAACAACGAAA  *R*: TAGGGAAGTGAAGAGGAGAAT | Nitrate transport | Root |
| *NR* | F: ACAGGCTTCTCCAGATACATTGGCG  R: AACTGAACCATGGGTGGTGGGC | Nitrate reduction | Leaf |
| *AMT* | *F*: AGATGAACTTGCGGGTATGG  *R*: TTGGAGTAGGAGTGGAAGAAGAA | Ammonium transport | Root |
| *GS* | *F*: GTCCAAGTGGTCTTTAAGCACAAA  *R*: GAGTAAAATCAAGAAGCAACCATGTTA | Glutamine synthesis | Leaf |
| *GOGAT* | *F*: GCACTGTTGCATCCGGG  *R*: TCCTGCATGTTTGATGGAACTT | Glutamate synthesis | Leaf |
| *pnp* | *F*:TGTCATCAGGCTTGCGGAA  *R*:ATCGCATTTTCGAGAGCGG | Housekeeping | Rhizobia |
| *β-*actin | *F*: CAGCCCACTGGATGTCTGTA  *R*: GTAGCAGCGCAAATTGAAGA | Housekeeping | Leaf/root |
