# Supplementary material for: Elevated CO2 Modifies N Acquisition of Medicago truncatula by Enhancing N Fixation and Reducing Nitrate Uptake from Soil
Source: PLoS One. 2013 Dec 5;8(12):e81373. doi: 10.1371/journal.pone.0081373 (PMC3855279; doi:10.1371/journal.pone.0081373)
Supplement: File S2 — Table S2: P values from two-way ANOVAs for the effects of CO2 level, M. truncatula genotype, and their interaction on the growth traits and foliar chemical components of alfalfa plants. (DOC) [file pone.0081373.s002.doc]

**Table S2.** *P* values from two-way ANOVAs for the effects of CO_2_ level, *M. truncatula* genotype, and their interaction on the growth traits and foliar chemical components of alfalfa plants.

| Kind of response | Dependent variable | *P* value | | |
| --- | --- | --- | --- | --- |
|  |  | CO_2_^1^ | Geno^2^ | CO_2_×Geno |
| Growth traits | Above-ground biomass | <0.001*** | <0.001*** | 0.005** |
|  | Below-ground biomass | 0.025* | <0.001*** | 0.002** |
|  | Biomass | <0.001*** | <0.001*** | 0.003** |
|  | Nodule numbers | 0.003** | <0.001*** | 0.162 |
| TNC^3^ contents | Foliar TNC | <0.001*** | 0.065 | 0.635 |
|  | Root TNC | 0.004** | 0.033* | 0.024* |
| N concentrations and Ntotal Yield | Foliar N | 0.603 | 0.033* | 0.119 |
|  | Root N | 0.016* | 0.004** | 0.002** |
|  | Ntotal Yield | <0.001*** | 0.002** | 0.003** |
| N metabolic enzymes | Foliar NR^4^ | <0.001*** | 0.177 | 0.124 |
|  | Root NR | <0.001*** | 0.051 | 0.167 |
|  | Foliar GS^5^ | 0.205 | 0.025* | 0.009** |
|  | Root GS | 0.904 | 0.004** | 0.019* |
|  | Foliar GOGAT^6^ | <0.001*** | 0.037* | 0.038* |
|  | Root GOGAT | 0.018* | 0.005** | 0.003** |
| ^1^Ambient CO_2_ vs. elevated CO_2_. ^2^ Three genotypes of alfalfa (Jemalong, *dnf1-1*, *dnf1-2*). ^3^The total non-structural carbohydrates. ^4^Nitrate reductase. ^5^Glutamine synthetase. ^6^Glutamate synthase. *<0.05, **<0.01, ***<0.001. | | | | |
