# Supplementary material for: Elevated CO2 Modifies N Acquisition of Medicago truncatula by Enhancing N Fixation and Reducing Nitrate Uptake from Soil
Source: PLoS One. 2013 Dec 5;8(12):e81373. doi: 10.1371/journal.pone.0081373 (PMC3855279; doi:10.1371/journal.pone.0081373)
Supplement: File S3 — Figure S1: The legume genes shown in this figure were tracked in the current study and are involved in N fixation, N uptake from soil, and N assimilation as indicated. The genes include: early nodule-specific protein 40 (ENOD), nodulation genes (nodF), nitrogen-fixing genes (nifH), nitrate transporter NRT1.1 (NT), nitrate reductase (NR), nitrate transporter NRT1.1 (NT), ammonium transporter protein (AMT), glutamine synthase 2 (GS2), and glutamate synthase (GOGAT). (DOC) [file pone.0081373.s003.doc]

**Figure. S1.** The legume genes shown in this figure were tracked in the current study and are involved in N fixation, N uptake from soil, and N assimilation as indicated. The genes include: early nodule-specific protein 40 (*ENOD*), nodulation genes (*nodF*), nitrogen-fixing genes (*nifH*), nitrate transporter NRT1.1 (*NT*), nitrate reductase (*NR*), nitrate transporter NRT1.1 (*NT*), ammonium transporter protein (*AMT*), glutamine synthase 2 (*GS2*), and glutamate synthase (*GOGAT*).

**
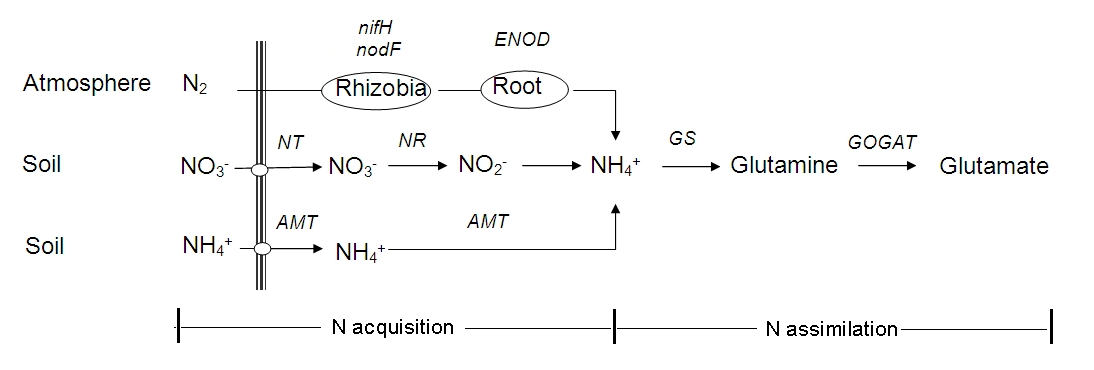
**
